# Fellow of the European board of ophthalmology glaucoma examination and diploma (FEBOS-Gl): update on 8 years of experience and future perspectives

**DOI:** 10.3389/fmed.2023.1163264

**Published:** 2023-06-16

**Authors:** Gordana Sunaric Mégevand, Alain M. Bron, Fotis Topouzis

**Affiliations:** ^1^Clinical Eye Research Centre Memorial Adolphe de Rothschild, Geneva, Switzerland; ^2^Centre Ophtalmologique de Florissant, Geneva, Switzerland; ^3^Department of Ophthalmology, University Hospital, Dijon, France; ^4^Eye and Nutrition Research Group, Centre des Sciences du Goût et de l'Alimentation, AgroSup Dijon, CNRS, INRAE, Université de Bourgogne Franche-Comté, Dijon, France; ^5^First Department of Ophthalmology School of Medicine, Aristotle University of Thessaloniki, AHEPA Hospital, Thessaloniki, Greece

**Keywords:** education, examination, glaucoma, European board of ophthalmology (EBO), European

## Abstract

Glaucoma is the leading cause of irreversible blindness worldwide. Early diagnosis and appropriate management of the disease are essential to avoid a significant impact on the quality of life of millions of patients and the socioeconomic impact on societies. Education is the hallmark of good medical care. The European Glaucoma Society (EGS) has dedicated significant efforts to provide means of improving education, training, and testing knowledge in the field of glaucoma. The Fellow of the European Board of Ophthalmology Subspecialty (FEBOS)-Glaucoma examination, introduced and organized yearly by the EGS since 2015 in collaboration with the European Board of Ophthalmology (EBO), has become a valuable tool for increasing overall knowledge in the field. Over the 8 years of experience, several updates and new projects have emerged around the examination to further increase the overall quality of education, training, and knowledge in the field of glaucoma in Europe, particularly in UEMS and associated countries. This article discusses in detail the various projects and measures introduced by the EGS.

## 1. Introduction

Glaucoma is the leading cause of irreversible blindness worldwide and its incidence will grow significantly in the future due to demographic changes related to prolonged life expectancy (1). Yet, too many glaucoma patients continue to encounter severe visual field loss during their lifetime or are bilaterally blind (2). Early diagnosis and appropriate management of glaucoma can decrease not only conversion from ocular hypertension (OHT) to glaucoma (3) but also lower significantly the progression of the disease, thus preserving health-related quality of life (4–6). Knowledge in the field of glaucoma is continuously evolving. In recent years, we have witnessed an abundance of new data extending from epidemiology, genetics, and advancements in technology to new medical and surgical therapy (7). Therefore, continuous updating of knowledge, education, and training in the field of glaucoma among ophthalmologists is the most important means available to enable appropriate and personalized management of the disease.

Thus, medical training, in general, depends on local and regional resources as well as on socioeconomic systems with significant heterogeneity in medical practices, since each country has its own teaching programs and its own medicolegal and administrative rules. In this regard, Europe is an excellent example of heterogeneity resulting in significant differences in patient care. To address this problem, the ophthalmology section of the European Union of Medical Specialties (UEMS), a professional organization was formed in 1958 that represents medical specialists in the European Union (https://uems.eu), established by the European Board of Ophthalmology (EBO) in 1992 (8). As an educational working body, the essential role of the EBO is to promote harmonization in education, training, and knowledge in ophthalmology in the UEMS and its associated countries (https://eusem.org/images/List_of_UEMS_Members.pdf). Over the past few decades, the EBO has continuously gained respect for successfully harmonizing education and training. Since 1995, one of its most important activities has been the yearly organization of the comprehensive European Board of Ophthalmology Diploma (EBOD) examination, which aims to ensure a minimum standard of knowledge in comprehensive, general ophthalmology among specialists and residents in UEMS (associated) countries (8). The Fellow of the European Board of Ophthalmology (FEBO) title awarded to successful candidates has become the hallmark of formal validation of the acquired level of knowledge in comprehensive, general ophthalmology.

While basic training in ophthalmology is very heterogeneous, subspecialty training, including, for some subspecialties, additional surgical training, is even more disparate between European countries, which may have a considerable impact on the quality of care. To address this problem, the EBO has more recently introduced the concept of specific subspecialty examinations awarding successful candidates with the diploma and title of Fellow of the European Board of Ophthalmology Subspecialty (FEBOS) (9). This project aimed to harmonize knowledge in various subspecialties across European countries. By setting clear standards of the knowledge and expertise to be tested during the FEBOS examination, the goal is to recognize the expertise and competence to be awarded through the FEBOS diploma. Although EBO remains the “umbrella organization” for all subspecialty FEBOS examinations and certifies the global guidelines, it has invited European subspecialty societies to actively take the lead in organizing and chairing FEBOS examinations within their subspecialty.

The European Glaucoma Society (EGS) in collaboration with EBO was the first European subspecialty society to actively take the lead in organizing the first subspecialty examination in 2015: the FEBOS-Glaucoma (FEBOS-Gl) examination.

This article aims to give a review and analysis of the 8-year experience with the FEBO-Gl examination and to inform on several new projects led by the EGS to improve the quality of the examination. It also provides details and updates on various procedural aspects of its organization ensuring transparency to future participating candidates as well as to the responsible leaders in glaucoma education.

## 2. The FEBOS-Gl examination: what changed over the past 8 years?

While the initial goals of the FEBOS subspecialty examination in glaucoma have been clearly described in a previous article and are still valid (10), experience over the past few years has shown that several parameters relevant for the achievement of the best quality of this project needed further consideration. Initially, the original goal was described as follows: “To increase homogeneity in subspecialty standards of training, knowledge, and practice across European countries. By introducing FEBOS examinations, the EBO aims to create clarity on the standards to be achieved and to provide a document recognizing expertise and competence. The FEBOS document will be a diploma given to ophthalmologists who have completed their subspecialty training and have passed the FEBOS examination” (10). Defined as such, this goal places the main emphasis on the harmonization of training and teaching, which has, indeed, been accomplished by providing detailed, standardized instruments: the EGS syllabus, logbook, and curriculum, which constitute a clear frame of the extent of theoretical and practical knowledge required to apply for the examination and the diploma (https://www.eugs.org/data1/EBO_curriculum.pdf). While the goal of harmonizing knowledge in glaucoma throughout Europe remains an essential task, new evidence has emerged from the consecutive yearly experiences of FEBOS-Gl examinations. It became clear that it was important to set a new standard regarding the difficulty and quality of the FEBOS-Gl examination and that the level targeted should represent a new benchmark for the assessment of knowledge. Unlike the European Board Ophthalmology Diploma (EBOD) comprehensive examination, which aims at ensuring a minimal standard of knowledge (11), the FEBOS-Gl examination should aim for “excellence in knowledge, professional aptitude and attitude,” and the FEBOS-Gl diploma should represent proof of the efforts expended by the successful candidates.

In order to reach this goal of setting a higher bar, various adjustments had to be made. The EGS has taken advantage of several important changes within its structure and identified four points of action in order to improve the overall quality of education, training, and knowledge in the field of glaucoma and subsequently the value of the FEBOS-Gl examination and diploma. These adjustments and changes can be summarized as follows: (1) to target the correct population of candidates fulfilling the predefined strict requirements for eligibility; (2) to improve training in the field of glaucoma; (3) to continuously update and improve the written and oral assessment tools; and (4) to ensure a comprehensive scoring system in order to provide consistency and transparency.

### 2.1. Targeting the correct population for the FEBOS-Gl examination

For the initial version of the FEBOS-Gl examination in 2015, the EGS established a document with specific requirements to be fulfilled by the candidates. One of the requirements concerned previous glaucoma training and stated as follows: “After graduation from your ophthalmology program, did you complete a minimum one-year glaucoma-focused training in a university hospital?” Although this statement provides clear guidance on the educational status of the eligible candidates, practical experience has shown that this requirement was sufficiently vague to lead to possible misinterpretation. Indeed, as worded, the statement encouraged some candidates who were interested in glaucoma but did not want to pursue it as their professional choice or as an exclusive subspecialization to apply despite having only a limited experience in the field. This resulted in large variability in basic knowledge, training, and experience and ultimately in variable individual scores in the examination since its introduction in 2015. Indeed, only two-thirds of the applications submitted between 2015 and 2022 were accepted since the candidates did not fulfill the criteria, and only 62% of those who took the examination passed it (Table 1).

**Table 1:** Recruitment and outcome of candidates for the fellow of the European board of ophthalmology subspecialty glaucoma examination.

**Year**	**Applications**	**Eligible candidates**	**Pass *n* (rate)**
2015	19	12	8 (67%)
2016	12	7	4 (57%)
2017	12	9	5 (56%)
2018	10	7	5 (71%)
2019	8	7	3 (43%)
2022	14	11	8 (73%)
Total	75	53	33 (62%)

In 2020 and 2021, the FEBOS-Gl examination could not be held due to the COVID-19 pandemic.

**Figure 1:**
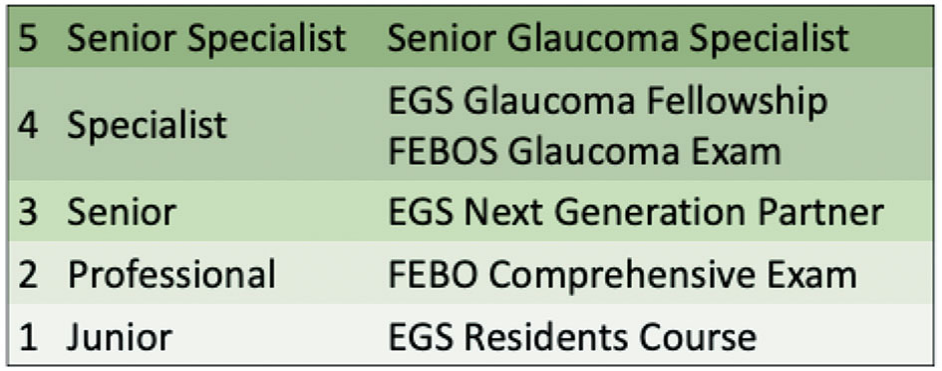
The European glaucoma society career scheme for glaucoma specialists. EGS, European Glaucoma Society; FEBO, Fellow of the European Board of Ophthalmology; FEBOS, Fellow of the European Board of Ophthalmology Subspecialty.

The ideal target population has thus become, although not exclusively, the next-generation partners (NGPs) of the EGS, an EGS-Special Interest Group (SIG) created in 2016 with well-defined goals and activities within the EGS (https://www.eugs.org/eng/SIGngpmission.asp). This SIG comprises young European specialists in ophthalmology with a particular interest in and professional focus on glaucoma, who have received extensive glaucoma training in Europe or a structured glaucoma fellowship overseas. Today, the SIG counts ~80 members who are invited every year to participate in a specific event organized by the EGS for NGPs exclusively, consisting of a personal development program based on five different modules (https://www.eugs.org/data1/NGP-manifesto.pdf). Therefore, in the EGS career scheme (Figure 1), the NGP status seems to be naturally associated with the FEBOS-Gl examination, in that NGPs are suitable candidates. To date, 16 NGPs have successfully applied and passed the FEBOS-Gl examination.

### 2.2. To improve training in the field of glaucoma

While encouraging NGP members to take the FEBOS-Gl examination aided in the selection of candidates to some extent, it did not solve the problem of the heterogeneity in the quality of glaucoma training required to achieve the predefined level of knowledge and experience prior to the examination. Unlike some Anglo-Saxon countries with their longstanding tradition of organizing fellowships (FSs), European countries, in general, have only a few official subspecialty training programs or fellowships or none at all. While many so-called clinical fellowships are advertised in excellent teaching centers, they are mostly linked to the reputation of an institution and/or a person in the field who is willing to provide knowledge and training in the given subspecialty without an officially set framework or a final certificate. Indeed, most of the candidates for the FEBOS-Gl examination reported some type of glaucoma training, either within their mandatory residency or as a research project varying in terms of duration or location and with mentors of their own choice but not with a standardized and structured framework. Indeed, often the term “glaucoma subspecialty training or fellowship” is misleadingly used for what is in reality an *observership*, a non-remunerated visiting position, of variable and undefined timeline with no official diploma. Although this type of experience may provide some level of valuable education, it remains without personal involvement in the medical or surgical activity and in the respective duties of the trainee.

There are two main barriers to promote proper FS Europe-wide programs involving the transnational mobility of the trainees. The first is the heterogeneity of medical, legal, and administrative systems throughout the UEMS and associated European countries, which limit access to active clinical and, more importantly, surgical patient management to fellows who come from other countries than the teaching center. Indeed, even if the candidate has a national diploma issued in one of the UEMS or associated countries, it is mostly impossible to get a license to practice in the country of the host teaching center. This is a particular issue for surgical activities that remain inaccessible.

The second barrier is the linguistic diversity throughout Europe. This represents a real problem that limits interaction with patients and their management and makes communication with colleagues very difficult or even impossible.

While the EGS in the past offered glaucoma training programs in various centers in Europe, these aforementioned barriers most often did not allow candidates to perform patient-related procedures (clinical examinations, surgery, and laser procedures) necessary for a complete clinical and surgical training, thus transforming this experience into a research fellowship.

To overcome these barriers, the EGS has started by defining the term “fellowship” and has provided details on the content and structure of each different type of fellowship (https://www.eugs.org/eng/EGS_Fellowship_Observership_Definition.asp). Then, the EGS initiated a new ambitious project, the “EGS Europe wide Glaucoma Fellowship project” (https://www.eugs.org/eng/EGS_Fellowship.asp). This project intends to create, support, and validate official, well-defined, standardized, and structured fellowship programs on a national level in various EGS-accredited centers throughout Europe (UEMS and associated countries). This 1-year FS program is based on EGS standards defined by the syllabus, logbook, and curriculum and sets the framework for optimal training in the field of glaucoma. While this kind of framework guarantees standardization and harmonization in training and ultimately in overall knowledge in the field, it increases the possibility of clinical and surgical experience for each interested candidate throughout UEMS and associated countries. This project will represent an additional educational tool used by the EGS to enrich and complete the professional evaluation stepladder, allowing the trainee to continuously move upward, from a fellow position toward the FEBOS-Gl examination and diploma, and finally to a senior consultant or leading academic position. This will, on the one hand, satisfy and enforce the initial goal of harmonizing teaching and training and, on the other hand, will provide high-level training for future leaders in the field. Finally, it will increase the overall quality of glaucoma care in Europe.

### 2.3. Continuously improving the written and oral assessment tools

Following the rules of the EBO comprehensive examination, the FEBOS-Gl examination is divided into two parts: a written part consisting of 80 multiple choice questions (MCQs) (one stem followed by five answers, in total 400 questions of which each can be true or false) accounting for 50% of the overall score, and a viva voce (VV) partly based on clinical cases focusing on clinical and practical knowledge and professional aptitude and attitude accounting for the other 50% of the total score.

#### 2.3.1. The MCQs

In 2015, for the first edition of the FEBOS-Gl examination, the MCQs were harvested from the general EBO MCQs bank. These glaucoma-related questions came from previous EGS courses, organized around various EGS congresses and meetings. Depending on their educational content and difficulty, these courses could apply to the EBO and receive an official EBO accreditation through the European Network of Education and Training (ENET), an EBO working body (http://www.european-net.org/home/). The aim of the EBO accreditation is not only to ensure valid content for candidates who prepare for the EBO comprehensive examination but also for the EBO to obtain high-quality MCQs from course directors with questions related to the given content of the course. All these glaucoma-related MCQs were transferred to the EBO-EGS MCQs bank, updated, if necessary improved, and used for the FEBOS-Gl examination.

Since 2015, the EGS has been continuously feeding its own MCQ bank with new MCQs from various sources; they are provided by key opinion leaders in various subtopics, from EGS courses, or created by the EGS subspecialty examination committee members (https://www.eugs.org/eng/education_chair_action.asp). They are reviewed in detail by several members of this committee checking for quality, scientific content, structure, and difficulty. In addition, they are continuously adapted and enriched with new high-quality MCQs updated to current scientific knowledge. Once updated, corrected, dated, and validated, they are stored in the EGS MCQ bank only accessible with a password by the committee chair and co-chairs. Within the bank, the MCQs are classified by topics and subtopics, based on the EGS guidelines and the fellowship syllabus, on their difficulty, as well as on their last use in previous editions of the examination. Following the EBO rules for the EBO comprehensive examination, each MCQ can only be used once every 3 years, which demands a constant renewal in the MCQ bank.

The general rules for the MCQs are based on the EBO comprehensive examination, except for the number of MCQs. While the EBO contains 52 MCQs, the FEBOS-Gl examination consists of 80 MCQs with a stem followed by five answers, i.e., a total of 400 questions. This written part is conducted for 2.30 h. The candidate has three options: each response can be “true,” “false,” or “don't know.” This last option has been introduced by the EBO considering it is a valid choice for candidates and that it could decrease the number of wild guesses (8). In order not to penalize the candidate for this choice, a score of 0 is given for “don't know” answers, while a negative mark of −0.5 is given for incorrect answers; a positive mark of +1 is given for correct answers. The negative marking for incorrect answers was introduced in 2010 as a way to continuously improve the quality of assessment with a favorable effect on the statistical performance parameters. In the usual type of MCQs, a mark of −1 is given to wrong answers (12). The main reason for the EBO to reduce this mark to −0.5 is to avoid any negative influence on the confidence of candidates during the EBO examination (8). While this probably does not represent a relevant issue for the candidates of subspecialty examinations, who are more mature in their professional experience, the same rule has been kept for the FEBOS-Gl examination.

#### 2.3.2. Viva voce

The viva voce part consists of three distinct oral examinations of 20 min each (in total 1 h per candidate) conducted by three separate juries (two examiners per jury). The examiners are EGS members, professionally active in the field of glaucoma and appointed to this task based on their experience in academic teaching and training. This oral part is organized around clinical cases on diagnosis as well as medical and surgical management incorporating most items of the EGS syllabus including theoretical knowledge of the recent landmark literature in the field. This oral part of the examination focuses more on the assessment of higher-order reflection and of the professional attitude and experience of candidates. Testing surgical skills is not part of the FEBOS-Gl examination; however, in-depth knowledge of pre-, peri-, and post-operative care around glaucoma surgery is mandatory and is part of both the written and oral examinations. Practically, each examiner presents 1–3 clinical cases for 10 min and discusses with the candidate the specific management, while the other examiner takes careful notes of the candidate's responses.

Based on the new rules adopted by the EBO for the VV part in the EBO comprehensive examination since 2018, the EGS has been continuously updating its own VV bank based on the same principle. In brief, this consists of assembling a large number of well-documented, representative but varying clinical, medical, and surgical glaucoma cases, structured in the same standardized way and adapted to the targeted population with the expected high(er) level of knowledge. The goal is to render the VV examination as objective, structured, and comparable as possible regardless of the jury, its expertise, and its background. By providing all candidates with the same set of high-quality, equally structured clinical cases, supported by precise keywords for the intention of the jury, this oral examination offers the highest quality and transparency and, even most importantly, the highest objectivity in assessment.

### 2.4. Statistical evaluation of the scoring system

The EBO has created a well-defined statistical method for analyzing the scores of the EBO comprehensive examination, described in detail elsewhere (11). For the first three sessions of the FEBOS-Gl examinations (2015, 2016, and 2017), the same rules were applied. In summary, the process for calculating the MCQ score until 2017 for both examinations consisted of calculating the average total score for all candidates and the standard deviation. Initially, total scores were converted into a scale of 1–10 based on a predefined conversion table, but since 2017, scores have been converted into a linear scale with a score of 4–10 between the minimum observed test score (equal to 4) and the maximum observed test score (equal to 10). The pass mark for the written part (pass mark equal to 6, with 4 and 5 being failure) was fixed based on the application of a norm-referenced pass mark, which means that the candidate's successful score should be above or equal to the average MCQ score of all candidates minus 1 standard deviation (SD). This rather generous assessment, adopted by the EBO for the EBO comprehensive examination, offers the advantage of (1) maintaining a stable passing rate (~90%) and (2) not jeopardizing the candidate's chance of passing if the examination turns out to be more difficult. If the score of the MCQ is lower than the pass mark of 6, the candidates could compensate for it provided they do not fail in any of the three sections of the VV part and have obtained a final score equal to or >6. The software tool for the processing of the examination sheets for the EBOD and the FEBOS MCQ scores is Speedwell MultiQuest, provided by the EBO (http://www.speedwellsoftware.com/).

In 2017, the EGS examination committee supported by the EGS executive board and approved by the EBO decided that the calculation used and very well suited for the EBO comprehensive examination was not suited for the level of the FEBOS-Gl examination, which targets a more specific group of ophthalmologists, aiming at “excellence in knowledge” rather than ensuring minimal knowledge as in case of the EBO comprehensive examination. Indeed, with the former system of calculating MCQ scores, a low total mean score for all candidates (e.g., <50% of correct MCQ responses) would still allow for a bad result, being 1 SD below the average score, to achieve a passing mark (passing mark of 6). Therefore, it was suggested that candidates of the FEBOS-Gl examination should have a fixed percentage of correct answers to a total of 400 questions in order to get a passing mark of 6. Candidates may still compensate for a score of 5 in the MCQs, provided they do not fail any of the three sections in the VV part and have obtained a final score equal to or >6. However, a score of 4 or 5 in one or more of the three VV sections, even with a high score in the MCQs, is a complete failure and cannot be compensated by any other score. This strict scoring system based on a given predefined percentage of correct answers has positive but also negative points. The positive side is complete transparency in analyzing the scores and comparability between candidates from different sessions. The negative side is the fact that the scores would not be adapted to the difficulty of the examination. For example, if the MCQs were more difficult, the scoring would penalize candidates and jeopardize their passing rate in the given examination. While this argument on the variability of the difficulty could be valid, experience has shown that good candidates usually had a percentage of correct answers in the MCQs of over 60%. In order to fix the adequate percentage of correct responses in MCQs, two scenarios were studied: a 60% or a 70% correct response rate for a passing mark of 6. Interestingly, with the 70% correct response rate, all candidates of a given session would be penalized and would have failed the examination, while a calculation based on a 60% correct response rate seemed to be more adequate. Therefore, it has been decided since 2017 to adopt consistently the rule of 60% of correct answers on MCQs as the prerequisite for obtaining the passing mark. This rule will be used for all FEBOS-Gl MCQ examinations regardless of the difficulty of the questions. Indeed, a dynamic range that would change every year depending on the difficulty of the examination and the quality of the candidates, as is used in the EBO comprehensive examination, would not award better-than-average knowledge but would potentially lead to a regression to the mean.

In summary, the predefined and stable scoring system allows for better quality and more objectivity in the assessment process. The predefined score of 60% correctly answered MCQs for reaching the passing mark of 6, the 50/50 rule accounting for the MCQs and VV, possible compensation for a score of 5 in the MCQs, provided a final score equal to or >6 is obtained in all three VV sections, and the mark of 4 or 5 in either the MCQ or any of the three VV being a definite failure are rules that provide transparency, consistency, and ultimately credibility to the FEBOS-Gl examination. They enable a better comparison to be made between candidates from different sessions, allow us to set a clear standard, and finally clarify expectations for candidates and trainers.

Before 2022, paper material was used both by the candidates to give their answers to the MCQs and by jury members for the VV part to forward their marks to the statistician in charge of the final calculation. This needed substantial time. In 2022, tablets were successfully used, whereby the MCQ responses of each candidate and the marks of each jury member were forwarded to a server and could be handled immediately. Therefore, these results could be reviewed and discussed by the examination jury and the EGS Examination Committee directly after the end of the examination. This procedure is of great value. First, it gives direct insight into the quality of the examination and the quality of candidates within a few minutes after the completion of the examination, thereby allowing the jury to have an open and direct discussion and evaluation. Second, this procedure gives insight into useful details and parameters to be changed or improved if necessary in future examinations.

More recently, in honor of Peter Watson, who described in 1968 how to perform a trabeculectomy, which is still the most widely performed surgical intervention in glaucoma worldwide, the EGS has introduced the Peter Watson Medal, an award offered to the highest scoring candidate(s) in the FEBOS-Gl examination at each session.

## 3. Discussion

The EGS is the first European society to have introduced a subspecialty examination. This confirmed the leading educational role of EGS in Europe and enforced its legitimate authority in providing education in the field of glaucoma. The EBO continues to have responsibility for harmonizing assessment conditions and assessment structures of FEBOS examinations, taking into account the Glasgow Declaration of the UEMS Council for European Specialty Medical Assessments (10).

Since its first edition in 2015, the FEBOS-Gl examinations have been held every year except for 2020 and 2021 because of the general lockdown and travel restrictions caused by the COVID-19 pandemic. To date, 33 candidates have passed the examination and obtained the FEBOS-Gl diploma and are thus entitled to add the FEBOS-Gl title to their official professional name (Table 1). With the rigorous requirements and the quality and difficulty of the examination, it has become obvious that most of the successful candidates are well-established glaucoma specialists, some active in leading academic positions with the responsibility of heading glaucoma departments and providing glaucoma training to residents and fellows. Therefore, today, the FEBOS-Gl title and diploma have become the hallmark of formal validation and proof of excellence in knowledge and expertise in the field of glaucoma.

The FEBOS-glaucoma exam is, to the best of our knowledge, the only glaucoma subspecialty exam addressed to specialists with experience and after specific training in this subspecialty.

Since the successful organization of the FEBOS-Gl exam, the EBO has strengthened its will to motivate other European societies to engage in subspecialty exams. Thus, since 2017, the ESCRS and since 2018, the European Pediatric Society are organizing yearly their FEBOS subspecialty exams following the setup and structure of the FEBOS – Gl.

The ophthalmology Board exam in the US, the surgery-oriented exam in Switzerland, and the ICO exam have different goals and aims. These exams provide certifications that attest that a doctor has superior knowledge and skill in general ophthalmology or ophthalmological surgery but not necessarily in a subspecialty.

The EGS is continuously making significant efforts in updating and introducing new tools to improve education in the field of glaucoma in general and in increasing the level and value of the FEBOS-Gl examination/diploma in particular. The continuous and regular update of the EGS guidelines, as the basis of knowledge, is now in its fifth edition (13). The EGS syllabus, logbook, and curriculum (https://www.eugs.org/eng/EGS_Fellowship_Observership_Syllabus.asp) are also continuously updated to comply with recent scientific advancements. The introduction of the new Europe-wide fellowship program will allow candidates to apply for a complete and structured FS in their own country in EGS-accredited centers proving the guarantee of quality and standardization. Finally, ongoing efforts to improve the overall quality of the structure and content as well as the scoring system of the FEBOS-Gl examination will further improve transparency and add value to the assessment of knowledge in the field and contribute to strengthening the value of this diploma.

## 4. Conclusion

Ongoing and future projects of the EGS aim at continuously strengthening its main goal as stated in the fifth edition of the guidelines: “The goal of care for people with or at risk of glaucoma is to promote their best possible wellbeing and quality of life with minimal glaucoma-related visual disability within a sustainable healthcare system” (13). This can only be achieved by continuous careful programming, support, and evaluation of education, teaching, and training. Let us heed the words of Confucius: “If you think in terms of a year, plant a seed; if in terms of 10 years, plant trees; if in terms of 100 years, teach the people.”

## Data availability statement

The original contributions presented in the study are included in the article/supplementary material, further inquiries can be directed to the corresponding author.

## Author contributions

GS contributed to the conception, design, and writing of the manuscript. AMB and FT reviewed and edited the manuscript. All authors contributed to the manuscript revision, reading, and approval of the submitted version.
